# Adhesion of *Porphyromonas gingivalis* and Biofilm Formation on Different Types of Orthodontic Brackets

**DOI:** 10.1155/2012/471380

**Published:** 2012-01-18

**Authors:** William Papaioannou, Athanasios Panagopoulos, Haroula Koletsi-Kounari, Efterpi Kontou, Margarita Makou

**Affiliations:** ^1^Department of Preventive and Community Dentistry, School of Dentistry, University of Athens, 115 27 Athens, Greece; ^2^Laboratory for Microbiology, Department of Periodontology, School of Dentistry, University of Athens, 115 27 Athens, Greece; ^3^Department of Orthodontics, School of Dentistry, University of Athens, 115 27 Athens, Greece

## Abstract

*Objectives*. To examine the interaction between *Porphyromonas gingivalis* and 3 different orthodontic brackets *in vitro*, focusing on the effect of an early salivary pellicle and other bacteria on the formation of biofilms. *Material and Methods*. Mono- and multi-species *P. gingivalis* biofilms were allowed to form *in vitro*, on 3 different bracket types (stainless steel, ceramic and plastic) with and without an early salivary pellicle. The brackets were anaerobically incubated for 3 days in Brain Heart Infusion Broth to form biofilms. Bacteria were quantified by trypsin treatment and enumeration of the total viable counts of bacteria recovered. *Results*. Saliva was found to significantly affect (*P* < 0.001) adhesion and biofilm formation of *P. gingivalis*, with higher numbers for the coated brackets. No significant effect was detected for the impact of the type of biofilm, although on stainless steel and plastic brackets there was a tendency for higher numbers of the pathogen in multi-species biofilms. Bracket material alone was not found to affect the number of bacteria. *Conclusions*. The salivary pellicle seems to facilitate the adhesion of *P. gingivalis* and biofilm formation on orthodontic brackets, while the material comprising the brackets does not significantly impact on the number of bacteria.

## 1. Introduction

The formation of the dental microbial biofilm is facilitated by areas where the initially, loosely, adhering bacteria are protected from removal forces. Orthodontic brackets and appliances may provide such protection and as such may affect further plaque maturation. Maturation allows for the necessary conditions so that pathogenic microorganisms may prosper. Indeed, the literature shows that orthodontic therapy with fixed appliances cause increased plaque accumulation [1] with increased concentration of mutans streptococci and lactobacilli [2, 3]. *In vitro* studies have shown that differences exist between the different types of brackets concerning the adhesion of bacteria, especially for species that are involved in caries [4, 5]. The role of saliva and the salivary pellicle in the adhesion process has led to differing results in these studies.

Apart from the relationship between orthodontic therapy and the increased risk of demineralization of teeth and caries formation [6], there is also an increased risk for inflammation of the gingiva [7]. A common reaction is the hyperplastic form of gingivitis, especially in patients with fixed orthodontic appliances that include the use of brackets. These reactions may be attributed to changes in the local microbiota [8]. In a recent study [9] 2 different types of brackets were examined *in vivo* concerning the reaction of the tissues and the formation of microbial plaque on their surface. It was concluded that the type of bracket might have a severe effect on periodontal indices and microbial composition. Self-ligating brackets were found to cause a much faster change towards anaerobic bacteria, the bacterial types that are connected to periodontal pathology.

Despite the often-encountered association of orthodontic therapy with periodontal pathology and the bacteria involved in gingival inflammation, there is no data available in the literature concerning the characteristics and dynamics of the adherence and retention of periodontopathic organisms on orthodontic brackets, especially under *in vitro* conditions. This is in sharp contrast with cariogenic bacterial species for which the literature contains a significant number of studies.

The aim of the present study was to examine the interaction between periodontopathic bacteria and orthodontic brackets *in vitro*. *P. gingivalis* adhesion and biofilm formation were examined for 3 different bracket types based on the base material, for example, stainless steel, ceramic, and plastic. Moreover, the effect of a salivary pellicle and other bacteria on the formation of the biofilms was examined.

## 2. Materials and Methods

Adaptations to a previous experimental protocol [5] have been made in order to examine not only the adhesion but also the formation of biofilms by strict anaerobes (for the most part) involved in periodontal disease.

Briefly, biofilms of *P. gingivalis* were allowed to form on brackets composed of 3 different materials. These surfaces were either with or without a salivary pellicle (coated *versus* uncoated brackets). The biofilms were monospecies, that is, made up of only *P. gingivalis* (Pg), or multispecies, *P. gingivalis* in combination with 3 other species (Pg+) (see below). After 3 days of growth the bacteria comprising the biofilms were harvested and the total number of viable bacteria were enumerated for each situation. A detailed account follows.

### 2.1. Bacterial Culture Procedures

Three laboratory strains of bacteria were used: *Fusobacterium nucleatum* (DSM 15643), *Streptococcus oralis* (DSM 20627), *P. gingivalis* (DSM 20709), as well as clinical isolates of *Actinomyces* spp. from the Periodontology Clinic of the Dental School of the University of Athens. All the bacteria were stored in sterile vials containing porous beads (Microbank, Pro-Lab Diagnostics) at −70°C.

Before the bacterial experiments on brackets, a few beads were taken with a sterile micrological loop from the frozen cultures of the 4 strains and were individually spread on blood agar plates (Blood Agar Base II; Oxoid, Basingstoke, England) supplemented with hemin (5 *μ*g/mL), menadione (1 *μ*g/mL), and 5% sterile horse blood and incubated. From these initial colonies, pure cultures were prepared on hard blood agar plates, further supplemented with 0.8% (w/v) Bacto Agar (Difco Laboratories, Detroit, Michigan, USA), to increase the hardness of the agar plates for easier collection of bacteria. These plates were anaerobically incubated (5% CO_2_, 10% H_2_, and 85% N_2_) for 5 days in jars (Oxoid, Basingstoke, UK) at 37°C. After 5 days the bacteria were collected and suspended in sterile phosphate-buffered saline (PBS) solution for the experiments. The final concentrations were set at 10^8^ bacteria per mL, for each species. These were adjusted by optical density measurements based on a previously calculated optical density/bacterial concentration gradient curve.

### 2.2. Preparation of Early Salivary Pellicle

On the first day of the experiments, saliva was selected from two healthy adults. They had not taken any medication during 3 months before the study and had no active caries or periodontal disease. Stimulated saliva was collected by chewing paraffin gum for 5 minutes and expectorating into a sterile plastic cup. The saliva was immediately clarified by centrifugation at 12,000 g for 20 minutes at 4°C and filtered using cellulose acetate membrane filters (pore size 0.22 *μ*m)

Half of all the brackets were prepared with the saliva in Costar 24-well-culture plates (Corning, NY, USA) for the formation of an early salivary pellicle. One mL of saliva was added to each well. They were incubated for 2 hours at 37°C after which they were removed and placed in new 24-well plates for the biofilm formation.

### 2.3. Biofilm Formation on Orthodontic Brackets

Metallic (stainless steel), ceramic (polycrystalline alumina), and plastic (polycarbonate) maxillary central incisor brackets (American Orthodontics, Sheboygan, WI) were included in the study. All brackets had a 0.018 inch slot. There were two different surface conditions. In the first case, for half of the brackets for each type no preparation was made before addition of bacteria, and, in the other case, the brackets were prepared with saliva as mentioned above. All situations (experiments) were examined together with common bacterial solutions.

In one part, 6 brackets of each type were placed in individual wells of a Costar 24-well culture plate, and Half of these (*n* = 3) were first prepared with saliva. A 2 mL Brain Heart Infusion Broth (BHIB) suspension of approximately 10^8^ per mL *P. gingivalis* was added to each well.

In a second part, *P. gingivalis *biofilm formation was examined in the presence of other species. Six (6) brackets of each type were placed in individual wells of a Costar 24-well culture plate, and half of these (*n* = 3) were first prepared with saliva. A 2 mL BHIB suspension of approximately 10^8^ per mL *P. gingivalis, F. nucleatum, S. oralis*, and *Actinomyces* spp. was added to each well.

For both parts, the brackets with the bacterial suspension were incubated at 37°C for three days in a jar (Oxoid, Basingstoke, UK) under anaerobic conditions. Every 24 hours fresh 2 mL of BHI was added (a total of 2 replenishments) in each well after decanting the old solution. Afterwards, the brackets were rinsed 2x carefully with PBS to remove any nonadherent bacteria.

### 2.4. Culture of Biofilm Bacteria

After the washing with PBS, the brackets with their adhering biofilm bacteria were placed in wells with 2 mL of 0.25% trypsin/EDTA and incubated at 37°C for 15 min with intermittent shaking for the detachment of the adherent bacteria. Serial dilutions were prepared after thorough pipetting and vortexing the initial solution. These dilutions were then plated by hand onto ETSA plates (Enriched Trypticase Soy Agar-ETSA-BBL Microbiology Systems, Cockeysville, MD, USA). For each bacterial species, serial dilutions of the initial concentration were also plated to control the number of bacteria added to each well. After 5 days of anaerobic incubation in jars at 37°C, the total number of viable counts (TVCs)/well (bracket) was determined. The unit of adhesion was considered to be the colony unit formed. For the second part, where four distinct bacterial species were involved, the isolated bacteria were characterized and identified based upon the colony morphology, Gram stain, and catalase activity.

### 2.5. Statistical Analysis

All statistical analyses were performed using the Data Analysis Toolkit of Microsoft Office Excel 2007. Use of 3 brackets per group was chosen in order that the experiment should have at least 80% power to detect, which is an acceptable power level, at 5% significance (statistical power was found at least 0.818 or 81.8%). Two-way ANOVA was used to test for the effects of salivary pellicle and bracket type, on the one hand, as well as of the effect of bracket type and mono- or multispecies biofilm formation. Only the mean total number of adherent *P. gingivalis* (as represented by the TVC) per type of bracket was statistically tested. All microbial data was log transformed. For all analyses *P* < 0.05 was considered statistically significant.

## 3. Results

**Figure 1:**
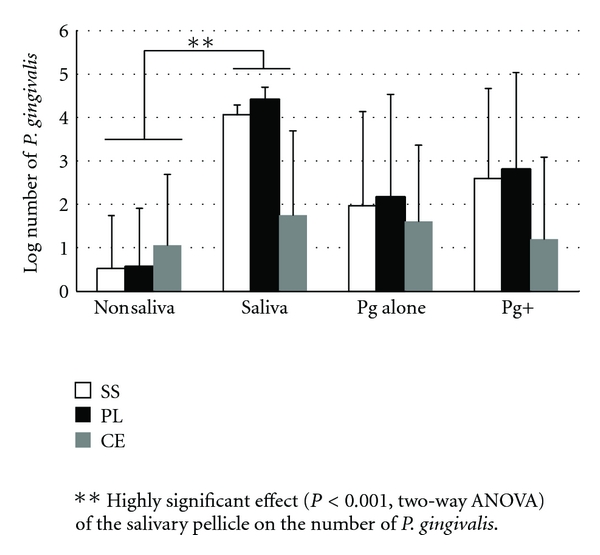
The mean log-transformed number and standard deviation of adhering *P. gingivalis* to the 3 types of brackets (*n* = 6 for each column). The first two sets of columns represent the effect of saliva coating (data is combined for the mono- and multispecies conditions). The other 2 groups present the mean numbers of *P. gingivalis* adhering alone or in combination with the other added bacterial species (Pg+) (data is combined for the nonsaliva and saliva coating conditions). (SS: stainless steel, PL: plastic, and CE: ceramic brackets).

**Figure 2:**
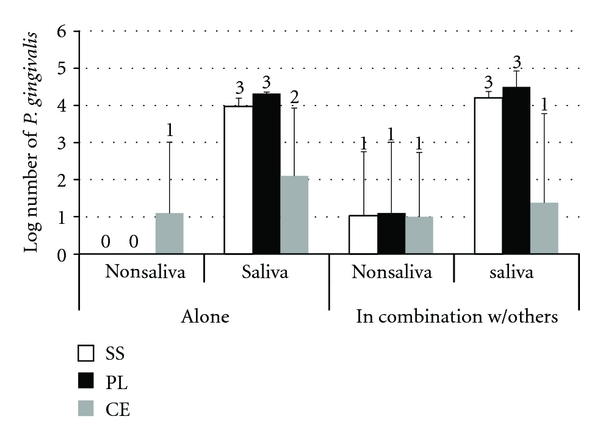
The mean log-transformed number and standard deviation of adhering *P. gingivalis* to the 3 types of brackets (*n* = 3 for each column). The data is split into the 4 distinct situations of non-saliva/saliva coating for *P. gingivalis* adhering alone or in combination with the other species. Numbers over each column represent the number of brackets with adhering *P. gingivalis.* (SS: stainless steel, PL: plastic, and CE: ceramic brackets).

From the results (Figures 1 and 2) the effect of saliva was found to significantly affect (*P* < 0.001) biofilm formation of *P. gingivalis* on the different types of brackets (Table 1). The number of bacteria increased greatly from the level of log 1 (noncoated brackets) to even above log 4 (for the coated). Moreover, the absence of a pellicle meant a higher number of brackets, in the monospecies biofilm group, that had even no biofilm formation (Figure 2). The type of bracket alone was not a significant factor; although some differences are apparent in the figure, the interaction of bracket type with the salivary pellicle, which had a very high level of significance (*P* = 0.009), would account for these.

When examining for the effect the type of biofilm, monospecies *P. gingivalis* or multispecies, no significant effect was detected (Table 2), regardless of the type of bracket. For the number of *P. gingivalis* (Figure 1), on stainless steel and plastic brackets there was a tendency for higher numbers of the pathogen to be found when considering multispecies biofilms. The multispecies biofilm allowed *P. gingivalis* to adhere and persist on at least 1 bracket for all 3 types, while without saliva and without the other bacteria no *P. gingivalis* was recovered on any of the stainless steel and plastic brackets (Figure 2).

**Figure 3:**
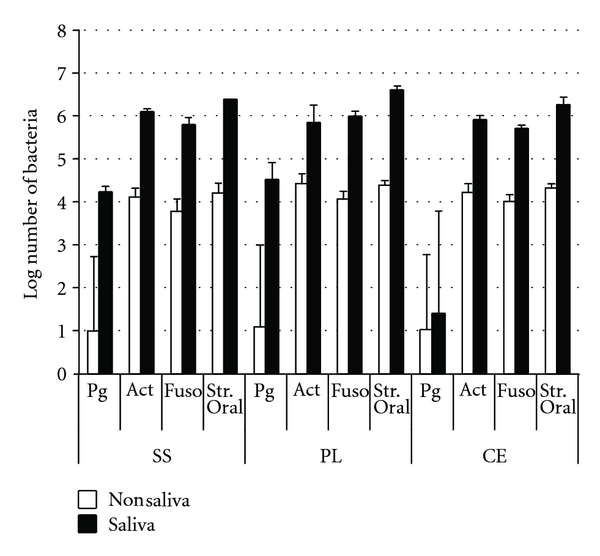
The mean log-transformed numbers of all the adhering bacteria for the 3 types of brackets, with and without saliva coating. For each column *n* = 3. All brackets had complete biofilms formed with all bacteria except for the noncoated brackets for which *P. gingivalis* was recovered in only 1 of every 3 types of brackets. (Brackets. SS: stainless steel, PL: plastic, and CE: ceramic brackets. Bacteria. Pg: *P. gingivalis*, Act: *Actinomyces* spp., Fuso: *F. nucleatum*, Str. Oral: *S. oralis*).

All the complementary bacteria (*S. oralis, F. nucleatum*, and *Actinomyces *spp.) that were added did adhere and form multispecies biofilms on all situations with or without saliva coating (Figure 3). Here too, the coated brackets, of each type, had higher numbers of all 3 bacteria. Although not statistically tested, the important differences can be appreciated.

## 4. Discussion

Orthodontic therapy, especially with fixed devices, causes a disruption in the homeostasis of the oral microbiota. This disruption is due to the increase in plaque retention [1], and as such a common effect is increase in species that can be considered as pathogenic to different oral tissues. Usually this concerns the hard tooth tissues and specifically an increase in the demineralization of enamel leading to an increase in white spot lesions or even eventually to caries lesions [10, 11]. However, inflammation of the gingiva is also a situation that is often encountered in the clinic [7]. This inflammation can be attributed to the increase in plaque build-up around the brackets, which in turn may result in the shift of the local microbiota towards a periodontopathic composition [8, 12].

Many studies have focused on the interactions of cariogenic bacteria, such as *S. mutans*, with different types of brackets [4, 5, 13–15]. However, only a few have looked at this interaction with periodontopathogens. The present study is the first to look at the adhesion of a periodontopathic bacterium and the formation of biofilms that may contain this microorganism on orthodontic surfaces, under *in vitro *conditions. Previously, a study examined the adhesion of lipopolysaccharides of *P. gingivalis* and *Escherichia coli* to two different bracket types [16], but not the bacteria themselves. More importantly, from retrieved brackets of orthodontic patients obtained during debonding and using the “checkerboard” DNA-DNA hybridization technique, Anhoury and coworkers [17] detected typical subgingival bacterial species, including *P. gingivalis*, on both ceramic and stainless steel brackets. The role they may play in periodontal inflammation is still unclear but nevertheless warrants further study.

The primary result of this study was that the salivary pellicle had a significant effect for development of both types of biofilm, *P. gingivalis* alone or multispecies (Figure 2). Previously, a significant effect of saliva was found for the adhesion of *S. mutans* on orthodontic brackets [5, 13, 14]. However, it was exactly the opposite than that seen for *P. gingivalis*; the pellicle led to a reduced number of adhering bacteria. There is a major difference in the study design, apart from the significantly different bacterial species examined, in that in the previous studies only adherence was examined, whereas here the bacteria had the time to form biofilms, either mono- or multispecies. In general though, *P. gingivalis* was not very competent to adhere to a surface and colonize it without the help of either saliva or other bacterial species. This is logical in terms of its characteristics that distinguish it as a late colonizer [18]. Moreover, these findings are in agreement with other studies examining the adherence of *P. gingivalis* to hard surfaces [19, 20].

It is clear that the surfaces that are coated with saliva provide the necessary receptors allowing the attachment of bacteria, which is a prerequisite for their further growth and biofilm formation. Indeed, Carlén and co-workers [19] have shown that both salivary proteins as well as plasma proteins (originating from the crevicular fluid) allow the attachment of bacteria, including *P. gingivalis*, to hydroxyapatite in both *in vivo* and *in vitro* situations. This explains the significantly higher numbers of bacteria seen in the biofilms of coated brackets.

The other factor which was examined and that could theoretically account for differences in attachment of bacteria and biofilm formation is the type of bracket. Although differences could be discerned, this was not found to be a significant factor, under *in vitro* conditions. Concerning the base material that the brackets were made of, plastic showed the highest number of bacteria in the biofilms, while stainless steel scored second and ceramic third. This may be due to differences in the adsorption of the proteins and other factors toward the surface that act as receptors, due to the differences in the chemical characteristics of the bracket material. This could logically result in differences in the pellicles that are formed which will in turn impact on the adhering bacteria. Just as with the differences with other studies concerning the effect of saliva and the salivary pellicle, the differences seen for the 3 different surface types are in disagreement with the studies examining *S. mutans* adhesion [4, 5, 21]. Of course the differences were not found to be statistically significant.

Apart from the surface characteristics that may affect the adhesion of bacteria, it is important to keep in mind the impact that the bracket design may have on the retention of bacteria, shielding them from removal forces. The importance of retentive surfaces of brackets was shown in recent study [9] where significantly higher retention of both aerobic and anaerobic bacteria was shown on self-ligating in comparison to conventional brackets after a week in the oral cavity. In the present study all brackets had similar design.

The formation of the dental plaque biofilm does not of course depend on one or even a limited number of species. Indeed we know that there is a complex interaction between different oral species as they adhere to the hard surfaces, with initial or early colonizers (primarily coccoidal bacteria), the intermediate or middle, and finally the late colonizers [18]. With this factor in mind the experiment was set up to include bacteria that could represent early and middle colonizers to facilitate the adhesion of the late-colonizing bacteria *P. gingivalis*. Indeed there did seem to be an effect of these bacteria, with a higher number of *P. gingivalis* being recovered, although this did not reach statistical significance. It is possible that this result could have been different if a larger number of samples could have been prepared and examined. Additionally, there is always the possibility that, by washing the brackets before harvesting the bacteria, as was performed in the present study, the more loosely adherent bacteria could possibly have been removed. This would be even more probable the more mature the biofilm is, but again further studies are necessary for in-depth investigation. Nevertheless, for the most part it would seem that the present findings agree with those pointing towards the importance of mutualistic biofilms for allowing *P. gingivalis* to persevere [20].

The fact that orthodontic brackets can harbor periodontopathic bacteria, as was seen in clinical studies, means that these devices can be considered a possible reservoir for these microorganisms, one that is in close proximity to gingival tissues and the sub-gingival region. For these reasons the interaction of orthodontic material with periodontopathic bacteria must be further evaluated to determine the impact they may have on the periodontal health of the individual undergoing orthodontic therapy.

## 5. Conclusions

1. Within the limitations of this study, saliva, and the surface pellicle that it forms, promotes the adhesion of *P. gingivalis* and biofilm formation on orthodontic brackets.
2. The type (material) of orthodontic bracket does not alter significantly the ability of *P. gingivalis* to adhere and form biofilms.

## Figures and Tables

**Table 1:** The effect of the salivary pellicle. Two-way ANOVA table for the effect of the presence of a salivary pellicle on the total *P. gingivalis* in the biofilms.

ANOVA						
Source of variation	SS	df	MS	*F*	*P* value	*F* crit
Effect of saliva	65.8670	1	65.8670	39.9604	**0.0000**	4.170877
Effect of bracket	7.9896	2	3.9948	2.4236	0.1058	3.31583
Interaction	18.3761	2	9.1881	5.5742	**0.0087**	3.31583
Within	49.4492	30	1.6483			

Total	141.6819	35				

**Table 2:** Effect of biofilm type. Two-way ANOVA table for the effect of the presence of other adhering bacteria (multispecies biofilm) on the total *P. gingivalis *in the biofilms.

ANOVA						
Source of variation	SS	df	MS	*F*	*P* value	*F* crit
Effect of biofilm type*	0.7377	1	0.7377	0.1692	0.6837	4.170877
Effect of bracket	7.9896	2	3.9948	0.9164	0.4109	3.31583
Interaction	2.1777	2	1.0888	0.2498	0.7806	3.31583
Within	130.7769	30	4.3592			

Total	141.6819	35				

*Mono- or multispecies biofilm.
